# The effects of collaborative care versus consultation liaison for anxiety disorders and depression in Denmark: two randomised controlled trials

**DOI:** 10.1192/bjp.2023.77

**Published:** 2023-09

**Authors:** Nadja Kehler Curth, Carsten Hjorthøj, Ursula Brinck-Claussen, Kirstine Bro Jørgensen, Susanne Rosendal, Anders Bo Bojesen, Merete Nordentoft, Lene Falgaard Eplov

**Affiliations:** Copenhagen Research Center for Mental Health, Mental Health Center Copenhagen, Copenhagen University Hospital, Mental Health Services – Capital Region of Denmark, Hellerup, Denmark; Copenhagen Research Center for Mental Health, Mental Health Center Copenhagen, Copenhagen University Hospital, Mental Health Services – Capital Region of Denmark, Hellerup, Denmark; and Department of Public Health, Section of Epidemiology, University of Copenhagen, Copenhagen, Denmark; Psychotherapeutic Clinic, Mental Health Center Copenhagen, Copenhagen University Hospital, Mental Health Services – Capital Region of Denmark, Copenhagen, Denmark; Copenhagen Research Center for Mental Health, Mental Health Center Copenhagen, Copenhagen University Hospital, Mental Health Services – Capital Region of Denmark, Hellerup, Denmark; and Institute of Clinical Medicine, Faculty of Health and Medical Sciences, University of Copenhagen, Copenhagen, Denmark

**Keywords:** Anxiety disorders, depressive disorders, primary care, randomised controlled trial, cognitive–behavioural therapies

## Abstract

**Background:**

Collaborative care (CC) and consultation liaison (CL) are two conceptual models aiming to improve mental healthcare in primary care. The effects of these models have not been compared in a Danish setting.

**Aims:**

To examine the effects of CC versus CL for persons with anxiety and depression in Danish general practices (trial registration: NCT03113175 and NCT03113201).

**Method:**

Two randomised parallel superiority trials for anxiety disorders and depression were carried out in 2018–2019. In the CC-group, care managers collaborated with general practitioners (GPs) to provide evidence-based treatment according to structured treatment plans. They followed up and provided psychoeducation and/or cognitive–behavioural therapy. The GPs initiated pharmacological treatment if indicated, and a psychiatrist provided supervision. In the CL-group, the intervention consisted of the GP's usual treatment. However, the psychiatrist and care manager could be consulted. Primary outcomes were depression symptoms (Beck Depression Inventory-II, BDI-II) in the depression trial and anxiety symptoms (Beck Anxiety Inventory, BAI) in the anxiety trial at 6-month follow-up.

**Results:**

In total, 302 participants with anxiety disorders and 389 participants with depression were included. A significant difference in BDI-II score was found in the depression trial, with larger symptom reductions in the CC-group (CC: 12.7, 95% CI 11.4–14.0; CL: 17.5, 95% CI 16.2–18.9; Cohen's *d* = −0.50, *P* ≤ 0.001). There was a significant difference in BAI in the anxiety trial (CC: 14.9, 95% CI 13.5–16.3; CL: 17.9, 95% CI 16.5–19.3; Cohen's *d* = −0.34, *P* ≤ 0.001), with larger symptom reductions in the CC-group.

**Conclusions:**

Collaborative care was an effective model to improve outcomes for persons with depression and anxiety disorders.

Anxiety disorders and depression are common mental disorders. The World Health Organization (WHO) has rated depression as the largest contributor and anxiety disorders as the sixth most significant contributor to disability globally.^1^ The majority of people diagnosed with anxiety or depression are treated in primary care.^2,3^ However, under-recognition and undertreatment are known problems in this setting.^4,5^ Collaborative care and consultation liaison are two conceptual models aiming to improve mental healthcare quality in primary care.^6^ Consultation liaison can be described as one or more mental healthcare providers assisting a primary care provider in the treatment of their patients.^7^ The contact between patients and the mental health specialist(s) varies across models and definitions.^7,8^ Aspects of consultation liaison can be an integral part of collaborative care interventions.^6^ Collaborative care can be described by (a) a multi-professional approach to treatment including a primary care provider and a minimum of one other provider (e.g. a care manager), (b) enhanced communication between providers, (c) treatment based on structured treatment plans and (d) scheduled follow-up of the patient's condition.^9,10^ The effects of collaborative care on depression and anxiety symptoms have been widely examined, primarily in the USA, showing that collaborative care improves outcomes for depression and anxiety disorders for up to 24 months, compared with treatment as usual.^9^ A meta-analysis including European studies only, for example from the UK and The Netherlands, has also shown positive effects of collaborative care on depression outcomes.^11^ The number of studies comparing consultation liaison with treatment as usual or collaborative care is limited, with inconsistent results.^7,8,12,13^ In a Danish setting, an evaluation of a consultation liaison intervention where general practitioners (GPs) could refer patients with suspected anxiety or depression to a psychiatrist for a single assessment showed no difference between groups in costs related to GP visits, but costs related to psychologist services in primary care and out-patient mental healthcare services were significantly lower in the intervention group than in a matched control group.^14^ Overall, the intervention was associated with a socioeconomic gain in the first year after referral.^14^ However, there was no evaluation of the participants’ symptoms.

As collaborative care is an organisational intervention, the effect of the intervention needs to be examined in other settings, including Scandinavian countries. Until the cluster-randomised Collabri studies were carried out in 2014–2017 examining collaborative care versus treatment as usual,^15^ no effect studies had been conducted in Denmark. Parallel with the Collabri studies, a Swedish study was carried out.^16^ As the Collabri studies were unsuccessful in recruiting the needed numbers of participants, in 2018 we began two new trials (the Collabri Flex trials), building on experiences from the Collabri studies.^17^ Low recruitment rates in the Collabri studies were linked to the cluster design, as the control group GPs in particular did not refer patients as anticipated. Therefore, the level of randomisation was changed from cluster to patient in the Collabri Flex trials. The risk of contamination bias was approached by adding a potentially contaminating element from collaborative care (mental health specialists supporting and supervising GPs) to the control group. The comparison group was therefore defined as a consultation liaison group, and the aim was to examine the effects of collaborative care versus consultation liaison. The hypothesis was that collaborative care would be more effective in reducing anxiety and depression symptoms than would consultation liaison.

## Method

### Study design

The methodology is presented in detail elsewhere.^17^ The two Collabri Flex trials were investigator-initiated randomised parallel superiority trials for anxiety disorders and depression respectively.

Recruitment of GPs occurred through GPs and care managers who had participated in the Collabri trials, a newsletter and a conference aimed at GPs.

The authors assert that all procedures contributing to this work comply with the ethical standards of the relevant national and institutional committees on human experimentation and with the Helsinki Declaration of 1975, as revised in 2008. All procedures involving human subjects/patients were approved by a Regional Ethics Committee in the Capital Region of Denmark (reference number H-16034303). The trials were registered 13 April 2017 at ClinicalTrials.gov (NCT03113175 and NCT03113201).

### Participants

Inclusion and exclusion criteria were assessed by the GP and/or a care manager. All participants were required to provide written consent to participate. Additional inclusion criteria in the depression trial were an ICD-10 diagnosis of depression (F32 or F33), the ability to speak Danish and age 18 years or older.^17^ In the anxiety trial, the same inclusion criteria applied except that instead of a depression diagnosis participants should have an ICD-10 diagnosis of social anxiety disorder (F40.1), panic disorder (F41.0), agoraphobia (F40.0), obsessive–compulsive disorder (F42), generalised anxiety disorder (F41.1) or post-traumatic stress disorder (F43.1).^17^ As recruitment rates were too low, obsessive–compulsive disorder and post-traumatic stress disorder were changed from being exclusion criteria to being inclusion criteria in the anxiety trial, and agoraphobia was added as an inclusion diagnosis after trial initiation. Persons were excluded if they were pregnant; had a diagnosis of dementia; had a severe medical condition that prevented participation; had a high risk of suicidality; had bipolar affective disorder; had a current psychotic condition, or severe alcohol or substance misuse; or had a referral to or need for mental health services in secondary care or from a private psychiatrist.^17^ Participants were also excluded if they had previously been included in the Collabri trials and had not yet participated in 15-month follow-up^15^ or were included in the Danish IBBIS trials, which tested an integrated vocational rehabilitation and mental healthcare intervention (by error, these criteria were not described in the trials’ registration). Participants were also excluded if they would not allow treatment from a psychologist or other similar treatment to be preceded by the Collabri Flex intervention if allocated to the collaborative care group.^17^

### Diagnostic assessment

Participants were referred by their GP. In the assessment of whether the diagnostic criteria for an anxiety disorder were fulfilled, GPs were encouraged to use the Anxiety Symptom Scale (ASS) according to recommendations.^18^ For the assessment of depression, GPs were randomised, as part of another study, to diagnose depression as they would normally do or mandatory use of the Major Depression Inventory (MDI) every time they suspected depression. Care managers used the Mini International Neuropsychiatric Interview (MINI) for DSM-IV disorders and ICD-10 specific questions to validate the diagnosis.^19^ Care managers discussed all the diagnostic assessments with a mental health specialist, who was either a psychiatrist or a specially trained psychologist. In the case of discrepancies between diagnoses, a project psychiatrist contacted the GP and together they decided the final diagnosis. Whether the participant was included in the anxiety or depression trial was determined by the final diagnosis.

### Randomisation and masking (‘blinding’)

Eligible participants were randomly assigned to receive either collaborative care or consultation liaison. A Collabri Flex team member carried out randomisation using a web application developed by the external provider OPEN – Open Patient data Explorative Network. Care managers informed participants of their allocation. The allocation sequence was computer-generated with variable block sizes hidden from research staff and those performing the randomisation during the project period. The allocation ratio was 1:1 in both trials. A stratification variable was previous pharmacological/psychological treatment for anxiety or depression. In the anxiety trial, the type of primary anxiety disorder was an additional stratification variable. The severity of depression was an additional stratification variable in the depression trial.

It was not possible to mask participants, their GPs or the Collabri Flex team to the participant's allocation. However, during the primary statistical analyses and while writing the main conclusion, researchers were masked to allocations.

### Interventions

#### Collaborative care

As described previously,^17^ the Collabri Flex collaborative care intervention was built on the Collabri intervention^15^ and adhered to the set of collaborative care criteria^9^ originally proposed by Gunn et al.^10^ A multi-professional approach to treatment was ensured by collaboration between a GP, a psychiatrist, a psychologist and a care manager. The GP had responsibility for the treatment and prescribed medication if needed. The GP could hand over the responsibility for parts of the treatment to the Collabri Flex team/psychiatrist. As a minimum, the GP had to agree on the treatment plan, any changes to this and prescribe medication. Care managers had equivalent to a year of training in cognitive–behavioural therapy (CBT) and a background of working in mental health services. Care manager sessions were predominantly provided in general practice or mental health service facilities. Each care manager collaborated with three to five GPs. Participants were offered treatment modalities according to diagnosis-specific treatment instructions and stepped care algorithms, providing stepwise intensification depending on the diagnosis and its severity. Treatment modalities were CBT (approximately 10–12 sessions), psychoeducation (independently for 3–4 sessions or as part of CBT) and medication. The psychoeducation and CBT were tailored to the participants; care managers could select from the diagnosis-specific therapeutic manuals. All participants were offered disease-specific written material; regular monitoring of the condition, including the use of structured instruments; monthly re-evaluation of the treatment plan; and the possibility of inviting a family member/friend to one or more care manager sessions. Care managers could also function as a link to the participant's other contact persons, for example in the job centre. According to model descriptions, the GP and their care manager should meet weekly to discuss participant progress. Case supervision of care managers by a psychiatrist and CBT supervision by a psychologist were scheduled to take place every second week. The psychiatrist supervision of GPs was planned to take place monthly, but was adapted to the GPs’ needs, where some received regular personal supervision/education and others preferred *ad hoc* supervision/education. Written communication between providers was secured through safe electronic systems.

Two fidelity reviews were conducted during the trial period.

#### Consultation liaison

The GP had overall responsibility for patient care and provided treatment as usual. The Danish College of General Practitioners and the Danish Health Authority have developed recommendations that could be used.^18,20–22^ For example, treatment could include support; psychoeducation; talking therapy; medication; or referral to a private psychologist, a private psychiatrist or mental health services. The GP could ask for advice and guidance from their care manager and the project psychiatrist regardless of the patient's allocation. Because of the risk of contamination bias when randomising on patient level, we included this potentially contaminating element of interaction between GPs and the mental health team in the control intervention, which we defined as consultation liaison.

### Outcomes

Self-reported outcomes were predefined and measured at baseline and 6 months after baseline, apart from satisfaction and support in personal recovery from providers, which were measured only at the 6-month follow-up. After trial initiation, an 18-month follow-up assessment was planned and initiated. Questionnaires were distributed and collected by the research team.

The primary outcome in the anxiety trial was Beck Anxiety Inventory (BAI) score.^23^ In the depression trial, the primary outcome was Beck Depression Inventory (BDI-II) score.^24^ Secondary outcomes were general well-being (WHO-5 Well-Being Index),^25^ general psychological symptoms (Symptom Checklist-90-Revised, SCL-90-R)^26^ and disability (Sheehan Disability Scale, SDS).^27^ BAI score was an additional secondary outcome in the depression trial, and BDI-II score was an additional secondary outcome in the anxiety trial. Explorative outcomes were measures of personal control (subscale from the revised Illness Perception Questionnaire (IPQ-R)^28^), self-efficacy (subscales from the Chronic Disease Self-Efficacy Scales: the Control/Manage Depression Scale and the Obtain Help from Community, Family, Friends Scale^29^), health status (three-level version of the EuroQol Five Dimensions Questionnaire, EQ-5D-3L^30^) and treatment satisfaction (Client Satisfaction Questionnaire, CSQ-8).^31^ A measure of support in personal recovery (INSPIRE)^32^ provided by the GP and care manager in the collaborative care group and by the GP in the consultation liaison group was also obtained. A register-based exploratory outcome was out-patient mental health contacts. In the trials’ online registration, measures of remission were not listed and the exploratory outcomes of sick-leave benefits and employment/education were not specified in detail. These were further defined in the protocol as the proportion of participants on sick-leave benefits at follow-up, the number of weeks on sick-leave benefits until follow-up, the proportion in employment/education at follow-up and weeks in employment/education until follow-up. Measures of adverse events were deaths from suicide/other, psychiatric and somatic admissions, somatic out-patient contacts, and the BAI and BDI-II. Other descriptive data of treatment provision were collected from Collabri Flex staff registrations and national registers.

### Statistical analyses

Sample size calculations had been done before the trial began.^17^ Based on a 4-point difference between means for both the BAI and BDI-II, a standard deviation of 12 for the BAI and 11 for the BDI-II, a statistical power of 80% and a significance level of 5%, 240 participants were required in the depression trial and 284 in the anxiety trial.

Differences in baseline characteristics were analysed using the *t*-test or Wilcoxon signed-rank test for continuous data, depending on distribution, or χ^2^ test for categorical data. Self-reported continuous outcomes were analysed using analysis of covariance (ANCOVA), and model assumptions were met when investigated for primary and secondary outcomes. Binary outcomes were analysed using logistic regression. Outcomes were adjusted for stratification variables and baseline values. Analyses were conducted according to intention-to-treat principles, and missing questionnaire-based end-point data were handled using multivariate normal regression imputations with 100 imputation sets and 10 burn-in iterations per set. The treatment effect estimates were derived from multiple imputations, apart from the registry-based outcomes, which had no missing data. The statistical analyses were performed in R version 3.2.1 for Windows.^33^ Before the primary analyses, a statistical analysis plan was developed. This plan also specified methods of analysis that had not been detailed in the protocol paper.^17^ Also, *post hoc* analyses were performed (Supplementary Box S1, available at https://dx.doi.org/10.1192/bjp.2023.77).

## Results

### Participants

**Fig. 1:**
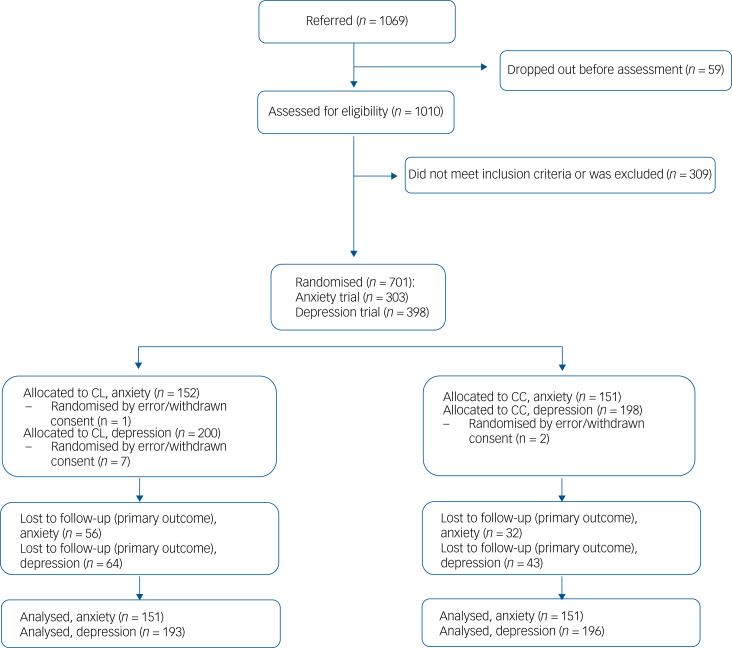
Flow chart showing patient referral and treatment allocation.

In total, 32 GP provider numbers (a provider number could include one or more GPs) agreed to take part; 29 GP provider numbers actively participated by referring patients to the trials. Participating patients were recruited between 15 January 2018 and 2 September 2019. In total, 302 participants with anxiety disorders (151 in each group) and 389 participants with depression (196 in the collaborative care group and 193 in the consultation liaison group) were recruited (Fig. 1).

Table 1 shows participants’ baseline characteristics. Around two-thirds in the depression trial fulfilled the criteria for moderate depression and around 25% fulfilled the criteria for severe depression. In the anxiety trial, about 48% had a diagnosis of panic disorder and/or agoraphobia and 30% had a generalised anxiety disorder. Comorbidity between anxiety and depression was seen in both trials (Table 1).

**Table 1:** Baseline characteristics

	Anxiety trial		Depression trial	
	Collaborative care (*n* = 151)	Consultation liaison (*n* = 151)	Collaborative care (*n* = 196)	Consultation liaison (*n* = 193)
Age, years: mean (95% CI)	35 (33–37)	36 (34–39)	37 (35–39)	40 (38–42)
Female, *n* (%)	112 (74)	98 (65)	128 (65)	123 (64)
**Marital status, *n* (%)**				
Not married or not living with a registered partner	113 (75)	111 (74)	151 (77)	142 (74)
Married or living with a registered partner	38 (25)	40 (27)	45 (23)	51 (26)
**Employment, *n* (%)**				
Employed	58 (38)	67 (44)	79 (40)	71 (37)
Studying	41 (27)	36 (24)	46 (23)	38 (20)
Unemployed	19 (13)	7 (5)	21 (11)	23 (12)
Receiving retirement/early retirement pension^a^	12 (8)	16 (11)	15 (8)	19 (10)
Receiving sickness benefits	14 (9)	17 (11)	28 (14)	31 (16)
No information	7 (5)	8 (5)	7 (4)	11 (5)
**Educational level, *n* (%)**				
Lower secondary education^b^	31 (20)	27 (18)	31 (16)	28 (15)
Upper secondary education	66 (44)	80 (53)	97 (49)	89 (46)
Short-cycle tertiary education, Bachelor or equivalent	27 (18)	23 (15)	55 (28)	54 (28)
Master, doctoral or equivalent	27 (18)	21 (14)	13 (7)	22 (11)
**Previous/current psychological/medical treatment, *n* (%)**				
Yes	73 (48)	71 (47)	82 (42)	81 (42)
**Previous psychiatric outpatient contacts, *n* (%)**^c^				
Yes	11 (7)	27 (18)	16 (8)	22 (11)
**Primary diagnosis, *n* (%)**				
Generalised anxiety disorder	45 (30)	45 (29)	−	−
Panic disorder/agoraphobia	72 (48)	71 (48)	−	−
Social anxiety disorder	26 (17)	26 (17)	−	−
Obsessive–compulsive disorder	8 (5)	9 (6)	−	−
Mild depression	−	−	16 (8)	15 (8)
Moderate depression	−	−	132 (67)	130 (67)
Severe depression	−	−	48 (25)	48 (25)
**Secondary diagnosis, *n* (%)**				
Anxiety diagnosis	35 (23)	52 (34)	59 (30)	68 (35)
Depression diagnosis	22 (15)	19 (13)	−	−
**Personality assessment, *n* (%)**				
Positive assessment of personality disorder (SAPAS >2)^d^	49 (34)	56 (40)	59 (33)	67 (38)
**Outcome measures, *n* (95% CI)**				
BAI	25.2 (23.8–26.6)	25.4 (24.0–26.8)	22.5 (21.0–24.1)	21.6 (20.4–22.8)
BDI-II^e^	22.2 (20.7–23.7)	21.3 (19.6–22.9)	32.1 (30.7–33.5)	30.4 (29.3–31.5)
SDS^f^	15.1 (14.0–16.2)	15.0 (13.8–16.2)	19.6 (18.7–20.5)	19.2 (18.3–20.0)
Personal control^g^	21.1 (20.4–21.7)	20.5 (19.8–21.2)	20.5 (20.0–21.1)	20.7 (20.2–21.2)
Self-efficacy (obtain help)^h^	6.4 (6.1–6.7)	6.4 (6.1–6.8)	5.4 (5.1–5.7)	5.6 (5.3–5.8)
Self-efficacy (symptoms)^i^	5.8 (5.5–6.0)	5.8 (5.5–6.1)	4.5 (4.3–4.8)	4.7 (4.4–4.9)
WHO-5^j^	36.5 (33.5–39.4)	38.2 (35.3–41.2)	22.5 (20.3–24.6)	23.9 (22.1–25.7)
SCL-90-R^k^	113.4 (106.3–120.5)	110.1 (102.5–117.6)	129.5 (121.5–137.5)	122.8 (116.8–128.7)
EQ-5D-3L	0.67 (0.65–0.70)	0.67 (0.64–0.70)	0.62 (0.59–0.65)	0.63 (0.61–0.66)

BAI, Beck Anxiety Inventory; BDI-II, Beck Depression Inventory-II; CC, collaborative care; CL, consultation liaison; EQ-5D-3L, three-level version of the EuroQol Five Dimensions Questionnaire; SDS, Sheehan Disability Scale; SCL-90-R, Symptom Checklist-90-Revised; SAPAS, Standardised Assessment of Personality, Abbreviated Scale; WHO-5, World Health Organization-5 Well-Being Index.

a. This category also includes other groups, such as those in job clarification.

b. This category includes those with missing data.

c. Based on contacts within the past 6 months.

d. Missing data in the anxiety trial: 7 in the CC-group and 10 in the CL-group. Missing data in the depression trial: 16 in the CC-group and 17 in the CL-group.

e. Missing data in the anxiety trial: 1 in the CL-group.

f. Missing data in the anxiety trial: 24 in the CC-group and 15 in the CL-group. Missing data in the depression trial: 29 in the CC-group and 25 in the CL-group.

g. Personal Control subscale from the Illness Perception Questionnaire-Revised (IPQ-R).

h. Obtain Help from Community, Family, Friends subscale from the Chronic Disease Self-Efficacy Scales.

i. Control/Manage Depression subscale from the Chronic Disease Self-Efficacy Scales.

j. Missing data in the depression trial: 1 in the CL-group.

k. Missing data in the anxiety trial: 1 in the CL-group. Missing data in the depression trial: 1 in the CL-group.

### Treatment

The collaborative care group's treatment lasted a little less than 4 months (Supplementary Table S1). On average, participants had almost nine care manager sessions, and most received CBT as the initial treatment modality. Across trials, around a fifth of the collaborative care participants had their treatment intensified, for example by adding medication to their current treatment or by being referred to more specialised care (Supplementary Table S1).

We do not have the same detailed information about treatment content in the consultation liaison group as for the collaborative care group. There was no significant difference between groups regarding the number of overall contacts with the GP (around seven contacts) (Supplementary Table S2a). However, a larger proportion in the consultation liaison group received one or more sessions of talking therapy with their GP (Supplementary Table S3) and had more contacts with a private psychologist in the follow-up period (Supplementary Table S2a).

### Communication between providers

In the collaborative care group, care managers sent written status notifications to GPs after re-evaluations and at the end of treatment. Although GPs had the possibility of discussing participants in the consultation liaison group with care managers, the option was not taken up to the same extent as for collaborative care participants (Supplementary Table S2b). Collaborative care participants in the anxiety trial were discussed *ad hoc* or during the biweekly treatment supervision sessions between care managers and the psychiatrist on average 0.3 times and participants in the depression trial were discussed on average 0.4 times (Supplementary Table S1). The psychiatrist was contacted *ad hoc* approximately twice a month by a GP. It was not assessed whether the contact concerned a collaborative care participant or a consultation liaison participant. In addition to this, some GPs met with the psychiatrist for supervision/education one or more times during the trial period.

### Fidelity

Fidelity reviews were conducted in September 2018 and October 2019. In the first fidelity review, the Collabri Flex team achieved good fidelity, and in the second review, fair fidelity to the Collabri Flex intervention model. Primarily owing to changes in the team composition as the project was nearing completion, the second review did not perform as well as the first. The project psychiatrist and CBT supervisor were available for fewer hours at the end of the trial than at its beginning, which was reflected in lower supervision frequency. Fidelity reviews also found that around half the treatment sessions were carried out in the GPs’ facilities. The model was developed to be flexible in terms of where the meetings were held. However, the care managers reported using much time on travel to different places because of this. Further, the frequency of meetings between GPs and care managers varied depending on the GP and was less than weekly in some instances.

### Outcomes

At the 6-month follow-up in the depression trial, we found a statistically significant difference in BDI-II scores (−5.6, 95% CI −7.3 to −3.9), with larger symptom reductions in the collaborative care group (collaborative care: 12.7, 95% CI 11.4–14.0; consultation liaison: 17.5, 95% CI 16.2–18.9; Cohen's *d* = −0.50, *P* ≤ 0.001). For the self-reported secondary and exploratory outcomes, we also found statistically significant differences in favour of the collaborative care group (Table 2 and Supplementary Fig. S1). At the 6-month follow-up in the anxiety trial, there was a statistically significant difference in BAI scores (−2.9, 95% CI −4.5 to −1.3), with larger symptom reductions in the collaborative care group (collaborative care: 14.9, 95% CI 13.5–16.3; consultation liaison: 17.9, 95% CI 16.5–19.3; Cohen's *d* = −0.34, *P* ≤ 0.001). There were several statistically significant differences between groups in the secondary and exploratory outcomes in favour of the collaborative care group (Table 3 and Supplementary Fig S2).

**Table 2:** Outcomes at 6-month follow-up in the depression trial

	Collaborative care		Consultation liaison				
Outcome measures (↑/↓ score = positive)^a^		Mean (95% CI)		Mean (95% CI)	Difference (95% CI)	*P*	Cohen's *d*
BDI-II ↓	12.7 (11.4 to 14.0)		17.5 (16.2 to 18.9)		−5.6 (−7.3 to −3.9)	≤0.001	−0.50
BAI ↓	12.4 (11.1 to 13.6)		15.9 (14.8 to 16.9)		−4.0 (−5.2 to −2.7)	≤0.001	−0.42
SDS ↓	9.3 (8.3 to 10.3)		12.6 (11.7 to 13.5)		−3.5 (−4.8 to −2.3)	≤0.001	−0.48
WHO-5 ↑	54.4 (51.3 to 57.5)		44.2 (41.4 to 47.1)		11.0 (7.1 to 14.9)	≤0.001	0.47
SCL-90-R ↓^b^	61.6 (55.2 to 68.0)		77.4 (71.2 to 83.5)		−19.4 (−26.6 to −12.3)	≤0.001	−0.35
Self-efficacy (symptoms) ↑^c^	6.6 (6.4 to 6.9)		6.0 (5.7 to 6.2)		0.7 (0.5 to 1.0)	≤0.001	0.39
Self-efficacy (obtain help) ↑^d^	6.7 (6.4 to 7.0)		6.1 (5.8 to 6.4)		0.7 (0.4 to 1.0)	≤0.001	0.29
EQ-5D-3L ↑	0.8 (0.8 to 0.8)		0.7 (0.7 to 0.8)		0.1 (0.0 to 0.1)	≤0.001	0.42
Personal control ↑^e^	23.2 (22.6 to 23.7)		22.3 (21.8 to 22.8)		1.0 (0.4 to 1.5)	≤0.001	0.23
INSPIRE-support ↑	74.1 (69.8 to 78.4)		49.4 (43.6 to 55.2)		24.6 (17.6 to 31.6)	≤0.001	0.82
INSPIRE-relationship ↑	87.0 (84.8 to 89.2)		67.5 (64.7 to 70.4)		19.5 (15.9 to 23.1)	≤0.001	0.95
CSQ-8 ↑	25.7 (25.1 to 26.4)		19.4 (18.8 to 20.0)		6.3 (5.4 to 7.2)	≤0.001	1.15
	***n*** (**%)**		***n*** (**%)**		**OR** (**95% CI)**		
In remission (BDI-II)^f^	116 (59)		87 (45)		1.9 (1.2 to 3.2)	0.008	−
In employment/education	125 (64)		107 (55)		1.3 (0.8 to 2.1)	0.220	−
Receiving sick-leave benefits	23 (12)		22 (11)		1.2 (0.6 to 2.2)	0.650	−
	***n***	**IR (95% CI)**	***n***	**IR (95% CI)**	**IRR (95% CI)**	***P***	
Weeks, employment/education	3038	15.5 (13.8 to 17.0)	2535	13.1 (11.4 to 14.7)	1.1 (1.0 to 1.2)	0.327	−
Weeks, sick-leave benefits	942	4.8 (3.7 to 6.0)	939	4.9 (3.7 to 6.2)	1.0 (0.8 to 1.5)	0.472	−
Contacts, psychiatric outpatient services	80	0.4 (0.2 to 0.8)	174	0.9 (0.5 to 1.5)	0.4 (0.2 to 1.1)	0.186	−
**Safety measures**^g^							
Contacts, somatic out-patient services	229	1.2 (0.8 to 1.7)	271	1.4 (0.9 to 2.0)	0.8 (0.5 to 1.4)	0.353	−
Days, somatic hospital admissions	10	0.05 (0.02 to 0.1)	11	0.1 (0.03 to 0.1)	0.7 (0.2 to 2.5)	0.496	−
Somatic hospital admissions	7	0.04 (0.01 to 0.1)	10	0.1 (0.02 to 0.1)	0.6 (0.2 to 1.7)	0.478	−
Deaths^h^	≤5		≤5				

BAI, Beck Anxiety Inventory; BDI-II, Beck Depression Inventory-II; EQ-5D-3L, three-level version of the EuroQol Five Dimensions Questionnaire; IR, incidence rate; IRR, incidence rate ratio; SCL-90-R, Symptom Checklist-90-Revised; SDS, Sheehan Disability Scale; WHO-5, World Health Organization-5 Well-Being Index.

a. For questionnaire data, estimates are based on imputed data adjusted for baseline values and stratification variables.

b. The SCL-90-R has a reference period of 2 weeks instead of 1 week.

c. Control/Manage Depression subscale from the Chronic Disease Self-Efficacy Scales.

d. Obtain Help from Community, Family, Friends subscale from the Chronic Disease Self-Efficacy Scales.

e. Personal Control subscale from the Illness Perception Questionnaire-Revised (IPQ-R).

f. Remission was defined by a score of 13 or less on the BDI-II.

g. Owing to Statistic Denmark's discretion rules (the register data provider), it was not possible to report on the number and days of psychiatric hospital admissions because of too few cases.

h. Owing to discretion rules, the exact numbers are not shown. Deaths did not include any suicides.

**Table 3:** Outcomes at 6-month follow-up in the anxiety trial

	Collaborative care		Consultation liaison				
Outcome measures (↑/↓ score = positive)^a^		Mean (95% CI)		Mean (95% CI)	Difference (95% CI)	*P*	Cohen's *d*
BAI ↓	14.9 (13.5 to 16.3)		17.9 (16.5 to 19.3)		−2.9 (−4.5 to −1.3)	≤0.001	−0.34
BDI-II ↓	10.2 (9.0 to 11.4)		13.2 (11.7 to 14.6)		−3.3 (−5.0 to −1.7)	≤0.001	−0.35
SDS ↓	8.5 (7.6 to 9.4)		9.8 (8.7 to 10.8)		−1.5 (−2.7 to −0.3)	0.018	−0.20
WHO-5 ↑	53.3 (50.2 to 56.4)		54.5 (51.4 to 57.6)		−0.4 (−4.5 to 3.6)	0.830	−0.06
SCL-90-R ↓^b^	63.6 (57.3 to 69.9)		76.5 (68.9 to 84.1)		−15.0 (−22.1 to −8.0)	≤0.001	−0.29
Self-efficacy (symptoms) ↑^c^	6.8 (6.6 to 7.1)		6.7 (6.4 to 7.0)		0.2 (−0.1 to 0.5)	0.286	−0.10
Self-efficacy (obtain help) ↑^d^	6.8 (6.5 to 7.1)		6.9 (6.6 to 7.2)		−0.1 (−0.4 to 0.3)	0.723	−0.05
EQ-5D-3L ↑	0.8 (0.8 to 0.8)		0.8 (0.8 to 0.8)		0.0 (0.0 to 0.1)	0.298	−0.12
Personal control ↑^e^	24.1 (23.5 to 24.6)		22.6 (22.1 to 23.1)		1.3 (0.6 to 1.9)	≤0.001	0.45
INSPIRE-support ↑	77.2 (72.9 to 81.6)		48.3 (41.9 to 54.7)		28.6 (21.2 to 36.0)	≤0.001	1.01
INSPIRE-relationship ↑	86.8 (84.3 to 89.4)		64.5 (61.7 to 67.3)		22.3 (18.5 to 26.1)	≤0.001	1.11
CSQ-8 ↑	26.9 (26.3 to 27.5)		19.1 (18.3 to 19.9)		7.8 (6.8 to 8.8)	≤0.001	1.31
	***n* (%)**		***n* (%)**		**OR (95% CI)**	***P***	
In remission (BAI)^f^	50 (33)		35 (23)		1.7 (0.9 to 3.2)	0.118	−
In employment/education	100 (66)		98 (65)		1.4 (0.8 to 2.6)	0.224	−
Receiving sick-leave benefits	13 (9)		10 (7)		1.3 (0.5 to 3.0)	0.593	−
	***n***	**IR (95% CI)**	***n***	**IR** (**95% CI)**	**IRR (95% CI)**	***P***	
Weeks, employment/education	2591	17.2 (15.4 to 18.9)	2606	17.3 (15.4 to 19.1)	1.0 (1.0 to 1.1)	0.304	−
Weeks, sick-leave benefits	291	1.9 (1.1 to 2.9)	372	2.5 (1.5 to 3.6)	0.6 (0.3 to 1.2)	0.235	−
Contacts, psychiatric outpatient services	55	0.4 (0.1 to 0.7)	199	1.3 (0.7 to 2.0)	0.2 (0.1 to 0.6)	0.039	−
**Safety measures**^g^							
Contacts, somatic out-patient services	149	1.0 (0.7 to 1.3)	192	1.3 (0.9 to 1.7)	0.8 (0.4 to 1.3)	0.301	−
Days, somatic hospital admissions	13	0.09 (0.01 to 0.2)	17	0.1 (0.04 to 0.2)	0.8 (0.2 to 2.0)	0.432	−
Somatic hospital admissions	6	0.04 (0.01 to 0.1)	14	0.1 (0.0 to 0.2)	0.4 (0.2 to 1.0)	0.207	−
Deaths	0		0				

BAI, Beck Anxiety Inventory; BDI-II, Beck Depression Inventory-II; EQ-5D-3L, three-level version of the EuroQol Five Dimensions Questionnaire with Three Levels; IR, incidence rate; IRR, incidence rate ratio; SCL-90-R, Symptom Checklist-90-Revised; SDS, Sheehan Disability Scale; WHO-5, World Health Organization-5 Well-Being Index.

a. For questionnaire data, estimates are based on imputed data, adjusted for baseline values and stratification variables.

b. The SCL-90-R has a reference period of 2 weeks instead of 1 week.

c. Control/Manage Depression subscale from the Chronic Disease Self-Efficacy Scales.

d. Obtain Help from Community, Family, Friends subscale from the Chronic Disease Self-Efficacy Scales.

e. Personal Control subscale from the Illness Perception Questionnaire-Revised (IPQ-R).

f. Remission was defined by a score of 9 or less in BAI.

g. Owing to Statistic Denmark's discretion rules (the register data provider), it was not possible to report on the number and days of psychiatric hospital admissions because of too few cases.

For both trials, no differences between groups were found in outcomes concerning employment/education and use of sick-leave benefits. However, there were fewer out-patient psychiatric contacts in the collaborative care groups compared with the consultation liaison groups, and for the anxiety trial, the difference was statistically significant (*P* = 0.039) (Tables 2 and 3). Tables 2 and 3 also show safety measures.

Sensitivity analyses showed no substantial differences from the main analyses (Supplementary Tables S4a–b), and only the extreme scenario where missing data in both groups in the anxiety trial were replaced with extremely low mean values showed no significant difference between groups. Exploratory subgroup analyses for those with mild depression in the depression trial and obsessive–compulsive disorder and social anxiety disorder in the anxiety trial showed no differences between groups. However, sample sizes were small for these groups (Supplementary Table S5). Further, in the anxiety trial, there was no difference between groups for those with no previous treatment experiences. Other subgroup analyses showed significant differences between groups, favouring collaborative care (Supplementary Table S5). Based on the effect sizes (Supplementary Table S5) and confidence intervals around treatment effects (not shown), there were no indications that the differences between effects were significantly different across subgroups, for example for those with/without positive screening for personality disorder.

## Discussion

### Summary of main findings

The collaborative care group received evidence-based and coordinated treatment according to an organisational model flexible in terms of treatment length, treatment location and GP involvement. At the 6-month follow-up, participants in the collaborative care groups were highly satisfied with the treatment and showed statistically significant improvements in symptom and functional levels compared with participants in the consultation liaison groups.

### Findings in relation to other research

There is limited research comparing collaborative care with consultation liaison. Two existing cluster-randomised trials reported inconsistent findings of either no significant differences in depression outcomes between collaborative care and consultation liaison or significant differences in favour of collaborative care.^12,13^ A reason why the Collabri Flex depression study shows more consistent findings in favour of collaborative care could be that the consultation liaison intervention is less intensive. In our trials, the mental healthcare team had no treatment-related contact with participants after the initial assessment. On the contrary, this was possible in both mentioned studies as the GP could refer to collaborating specialists.

Despite the literature being inconsistent, there are indications that consultation liaison is either as effective as treatment as usual^8^ or superior to treatment as usual on mental health outcomes in the short term.^7^ Substantial evidence shows that collaborative care is superior to treatment as usual.^9,11^ As treatment as usual may vary considerably across settings, we can only hypothesise that collaborative care would be superior to treatment as usual in a Danish setting.

### Strengths and limitations

Strengths of the trials were that the predefined sample sizes were reached and the studies add to the limited research comparing collaborative care with consultation liaison. The applied collaborative care model built on the model used in the Collabri trials^15^ and was a well-known model to many of the care managers and GPs, which might have prevented some initial operational challenges. The collaborative care model's implementation was further evaluated by two fidelity reviews, finding that most core model principles were carried out as intended. Additionally, allocation sequence concealment aimed to prevent selection bias and researchers were masked until primary conclusions were drawn.

Limitations might include a risk of detection bias, as most outcomes were self-reported and thus not masked. GPs recruited participants, thus it is unclear whether everyone eligible was invited to participate. Further, we do not know from these trials whether collaborative care is superior to treatment as usual in a Danish setting. In the anxiety trial, there were more participants in the consultation liaison group with a secondary anxiety disorder in addition to their primary diagnosis, which could reflect more severe anxiety conditions in this group. However, there was no statistically significant difference in anxiety symptoms at baseline. Nevertheless, we performed sensitivity analyses for both trials with adjustment for baseline differences, which did not change the interpretation of results.

### Implications

Based on the study results, collaborative care is recommended over consultation liaison. More than 70% of participants in the collaborative care groups completed treatment, and in the anxiety trial collaborative care also seemed to prevent further referrals to out-patient mental health treatment. The Collabri Flex approach is shown to be a useful model to improve outcomes for persons with anxiety and depression in Danish primary care. The results presented in this paper will, together with findings from 18-month follow-up, cost-effectiveness analyses and a study aiming to explore the conditions for implementation (e.g. through stakeholder interviews), guide whether further work towards implementation should be initiated.

## Data Availability

Any requests for data sharing should be directed to the corresponding author. Data that are shared on reasonable request will be anonymised.

## Supplementary material

For supplementary material accompanying this paper visit https://doi.org/10.1192/bjp.2023.77. click here to view supplementary material
