# Supplementary material for: The effects of collaborative care versus consultation liaison for anxiety disorders and depression in Denmark: two randomised controlled trials
Source: Br J Psychiatry. 2023 Sep;223(3):430–7. doi: 10.1192/bjp.2023.77 (PMC10895499; doi:10.1192/bjp.2023.77)

Supplementary material

Box S1.

Methods for post hoc analyses

Analyses not specified in the online protocol registration but specified in the statistical analysis plan (SAP)
According to the SAP, count measures/outcomes and safety measures should be analysed using negative binomial regression with robust standard error corrections in cases of severely skewed distributions with adjustment for stratification variables and baseline values. However, the models fitted poorly and Poisson regression with non-parametric bootstrapped confidence intervals was used instead. This model was also used analysing count data on GP, psychologist and psychiatrist contacts.
Subgroup analyses were carried out for stratification variables and a dichotomized SAPAS variable (screening for personality disorder). The same ANCOVA model specification as in the primary analyses was used, but with treatment effects estimated separately for each subgroup.
Sensitivity analyses were carried out with additional adjustments for unbalanced baseline variables (not register-based outcomes at baseline), observed data only, and extreme case analyses. In the two extreme case analyses, missing data in both groups were replaced with high mean values (90th percentile of the observed mean value), respectively, low mean values (10th percentile of the observed mean value) to assess the worst-case vs. best-case impact of missingness.
Effect sizes were estimated for the self-reported outcomes and not only the primary outcomes as specified. Effect sizes were estimated by dividing the mean difference by the pooled standard deviation at follow-up.

Analyses not specified in the online protocol registration or the statistical analysis plan
Analyses presenting proportions having contacts with a health care provider were analysed using chi^2 tests.
Sensitivity analyses testing three different models that take the nesting of patients into account were carried out: 1) geographical area (three different) and GP as a fixed effect, 2) geographical area and GP as a random effect (mixed model with random intercept), 3) with robust standard errors corrected for clustering within geographical area and GP.
In the anxiety trial, a sensitivity analysis taking the unequal distribution of psychiatric outpatient contacts at baseline into account using a bootstrapped linear model was also carried out (data not shown).

Table S1. Treatment information for participants in the collaborative care group

	Depression trial	Anxiety trial
**Contact between care manager and participant**		
Participants who had ≥ one treatment contacts [a], n (%)	193 (98)	146 (97)
Treatment length, mean days (months) per participant [b]	111 (3.7)	117 (3.9)
Treatment contacts, mean per participant [c]	8.7	8.7
**Contact between care manager and psychiatrist concerning participants**		
Contacts, mean per participant	0.4	0.3
**Initial treatment provided**		
Participants who received psychoeducation, n (%)	9 (5)	6 (4)
Participants who received CBT, n (%)	125 (64)	114 (75)
Participants who received CBT and medication, n (%)	54 (27)	25 (17)
Participants who received other treatment [d] or psychoeducation and medication, n (%)	8 (4)	6 (4)
**Intensification of treatment**		
Participants who stepped up one time or more, n (%)	46 (23)	29 (19)
**Treatment course**		
Participants who completed treatment, n (%)	142 (72)	119 (79)
Participants who were referred to specialist care before/during treatment, n (%)	26 (14)	18 (12)
Outpatient mental health care services, n (%)	13 (50)	8 (44)
Private psychologist/psychiatrist or other, n (%)	13 (50)	10 (56)
Participants who dropped out [e], n (%)	28 (14)	14 (9)

Abbreviations. CBT: Cognitive Behavioral Therapy.

[a] Includes participants who had minimum the first treatment planning consultation.

[b] Treatment length reflects the days from randomization date to registered date of treatment completed. The time from randomization to the first meeting with the care manager was on average 8.5 days.

[c] Includes contacts with a treatment purpose: the first treatment planning consultation (11% of total contacts, assumed average length: 60 min); other consultations/sessions incl. psychoeducation/CBT/CBT booster/other with/without monitoring/reevaluation (83% of total contacts, average length registered: 55 min); patient consultations with next of kin (4% of total contacts, average length registered: 55 min); other contact incl. monitoring/reevaluation face-to-face/phone/electronically and other (2% of total contacts, average length registered: 27 min). Percentages are provided across the two trials and do not include contact with a logistical purpose, e.g., calendar scheduling.

[d] Includes supportive sessions and no treatment.

[e] Includes drop out before or during treatment because of the patient's wish, missed contact, difficulties finding time or other reasons.

Table S2a. Contacts between participants and health care providers in primary care setting during 6-month follow-up (information from registers)

Depression trial						
	Collaborative care		Consultation liaison			
	N	IR (95% CI)	N	IR (95% CI)	IRR (95% CI)	*P*
GP (total)	1384	7.1 (6.3 to 7.8)	1477	7.7 (7.0 to 8.4)	1.1 (1.0 to 1.2)	0.336
GP (talking therapy)	187	0.95 (0.76, 1.16)	261	1.35 (1.12, 1.56)	1.37 (1.06-1.80)	0.090
Private psychologist	-	-	297	1.5 (1.1 to 2.0)	-	-
Private psychiatrist	-	-	81	0.4 (0.2 to 0.8)	-	-
**Anxiety trial**						
	Collaborative care		Consultation liaison			
	N	IR (95% CI)	N	IR (95% CI)	IRR (95% CI)	*P*
GP (total)	1047	6.9 (5.9 to 8.2)	1128	7.5 (6.6 to 8.3)	1.1 (0.9 to 1.3)	0.341
GP (talking therapy)	88	0.58 (0.4 to 0.76)	132	0.87 (0.69 to 1.09)	1.56 (1.0 to 2.4)	0.100
Private psychologist	13	0.09 (0.02 to 0.2)	78	0.5 (0.3 to 0.8)	6.9 (2.6 to 60.9)	0.010
Private psychiatrist	-	-	23	0.15 (0.0 to 0.3)	-	-

Abbreviations. IR: Incidence rate. IRR: Incidence rate ratio. Collaborative care is the reference group when reporting IRR. Some cells do not hold a number due to Danish protection rules concerning discretion.

Table S2b. Contacts between participants and the general practitioner about anxiety/depression and between GP and care manager concerning participants during 6-month follow-up (information from project registrations)

Depression trial						
	Collaborative care		Consultation liaison			
**Number of contacts between participants and the general practitioner**						
	N	IR (95% CI)	N	IR (95% CI)	IRR (95% CI)	*P*
GP (about anxiety/depression)	441	2.5 [a] (2.1 to 2.9)	517	3.1 [b] (2.7 to 3.5)	1.3 (1.1 to 1.5)	≤0.001
**Number of contacts between health care providers**						
	N	IR (95% CI)	N	IR (95% CI)	IRR (95% CI)	*P*
GP and care manager	269	1.37 (1.1 to 1.7)	16	0.1 (0.05 to 0.1)	0.08 (0.05 to 0.1)	≤0.001
**Anxiety trial**						
	Collaborative care		Consultation liaison			
**Number of contacts between participants and the general practitioner**						
	N	IR (95% CI)	N	IR (95% CI)	IRR (95% CI)	*P*
GP (about anxiety/depression)	247	1.9 [c] (1.4 to 2.4)	372	3.1 [d] (2.5 to 3.7)	1.9 (1.5 to 2.3)	≤0.001
**Number of contacts between health care providers**						
	N	IR (95% CI)	N	IR (95% CI)	IRR (95% CI)	*P*
GP and care manager	132	0.87 (0.7 to 1.1)	22	0.15 (0.1 to 0.2)	0.2 (0.1 to 0.3)	≤0.001

Abbreviations. IR: Incidence rate. IRR: Incidence rate ratio. Collaborative care is the reference group when reporting IRR. For GP contacts about anxiety/depression, full information was not available for all participants. Therefore, the total number of participants varied: [a] n=175, [b] n=168, [c] n=129, [d] n=120. Contacts between the GP and care manager could include face-to-face contacts, phone calls, e-mail or SMS contacts. It does not include written status notifications that care managers sent to GPs after each reevaluation and at the end of treatment for collaborative care participants. Contacts between care manager and GPs could include passing on treatment suggestions from the psychiatrist. Contacts between the care manager and psychiatrist concerning consultation liaison participants have not been registered separately as for participants in the collaborative care group (Table S1) and this data is thus not shown.

Table S3. Proportion of participants having contacts with a private psychologist, private psychiatrist or having talking therapy with a GP during 6-month follow-up

**Depression trial**				
		**Collaborative care**	**Consultation liaison**	
	**Number of contacts**	**Number of persons (%)**	**Number of persons (%)**	***P***
GP talking therapy	0	111 (57)	85 (44)	
	1-3	69 (35)	84 (44)	
	4+	16 (8)	24 (12)	0.039
Private psychologist	0	>180	144 (75)	
	1-4	≤5	19 (10)	
	5+	≤5	30 (15)	≤0.001
Private psychiatrist	0	>180	178 (92)	
	1-4	≤5	9 (5)	
	5+	≤5	6 (3)	<0.05
**Anxiety trial**				
		**Collaborative care**	**Consultation liaison**	
	**Number of contacts**	**Number of persons (%)**	**Number of persons (%)**	***P***
GP talking therapy	0	107 (71)	81 (54)	0.007
	1-3	37 (24)	62 (41)	
	4+	7 (5)	8 (5)	
Private psychologist	0	>140	133 (88)	<0.05
	1-4	≤5	8 (5)	
	5+	≤5	10 (7)	
Private psychiatrist	0	>145	>140	
	1-4	≤5	≤5	
	5+	≤5	≤5	>0.05

Abbreviations. GP: General practitioner.
Some cells do not hold a specific number due to Danish protection rules concerning discretion.

Table S4a. Sensitivity analyses

Depression trial					
	Collaborative care	Consultation liaison			
	Mean BDI-II (95% CI)	Mean BDI-II (95% CI)	Difference (Est)	*P*	Cohen's d
"Best case" analysis	10.5 (9.1 to 11.9)	12.5 (10.9 to 14.1)	-2.452	0.021	-0.19
"Worst case" analysis	16.2 (14.6 to 17.8)	21.1 (19.6 to 22.7)	-5.606	≤0.001	-0.44
Observed data	12.6 (11.0 to 14.3)	17.2 (15.3 to 19.2)	-5.702	≤0.001	-0.43
Adjustment for unequal baseline means	12.7 (11.4 to 14.0)	17.5 (16.2 to 18.9)	-5.571	≤0.001	-0.50
**Anxiety trial**					
	**Collaborative care**	**Consultation liaison**			
	**Mean BAI (95% CI)**	**Mean BAI (95% CI)**	**Difference (Est)**	***P***	**Cohen's d**
"Best case" analysis	13.2 (11.8 to 14.6)	13.1 (11.6 to 14.7)	0.116	0.908	0.007
"Worst case" analysis	17.9 (16.3 to 19.4)	21.3 (19.8 to 22.8)	-3.382	≤0.001	-0.36
Observed data	15.1 (13.5 to 16.8)	17.4 (15.4 to 19.3)	-2.730	0.019	-0.24
Adjustment for unequal baseline means	14.9 (13.5 to 16.3)	17.9 (16.5 to 19.3)	-2.953	≤0.001	-0.34

Sensitivity analyses are performed for the primary outcomes; BAI in the anxiety trial and BDI-II in the depression trial. Estimates for best case, worst case and adjustment for unequal baseline means are based on imputed data.

Table S4b. Sensitivity analyses using clustering

Depression trial			
**Model**	**Difference between means (Est)**	**SE**	***P***
Reference, without clustering of GP or area	-5.702	1.218	≤0.001
Cluster robust SEs for GP	-5.702	1.062	≤0.001
Fixed effect for GP	-5.396	1.256	≤0.001
Random intercept for GP	-5.702	1.218	≤0.001
Cluster robust SEs for area	-5.702	0.480	≤0.001
Fixed effect for area	-5.695	1.221	≤0.001
Random intercept for area	-5.702	1.207	≤0.001
**Anxiety trial**			
**Model**	**Difference between means (Est)**	**SE**	***P***
Reference, without clustering of GP or area	-2.730	1.156	0.019
Cluster robust SEs for GP	-2.730	1.172	0.020
Fixed effect for GP	-2.413	1.225	0.050
Random intercept for GP	-2.728	1.156	0.019
Cluster robust SEs for area	-2.730	1.148	0.018
Fixed effect for area	-2.748	1.167	0.020
Random intercept for area	-2.730	1.142	0.018

Abbreviations. GP: General practitioner, SE: Standard Error.
Sensitivity analyses are performed for the primary outcomes; BAI in the anxiety trial and BDI-II in the depression trial. Estimates are based on observed data. The proportion of variance explained (R^2) by GP clustering was 0.15 for BAI and 0.09 for BDI-II. The proportion of variance explained (R^2) by area clustering was 0.01 for both BAI and BDI-II.

Table S5. Subgroup analyses for the primary outcomes

Depression trial							
		**Collaborative care**		**Consultation-liaison**			
	**N**	**Mean BDI-II (95% CI)**	**N**	**Mean BDI-II (95% CI)**	**Difference (Est)**	***P***	**Cohen's d**
**Depression severity**							
Mild depression	16	11.5 (7.7 to 15.3)	15	16.7 (12.1 to 21.3)	-3.084	0.215	-0.64
Moderate depression	132	12.3 (10.8 to 13.8)	130	17.0 (15.5 to 18.6)	-5.013	≤0.001	-0.52
Severe depression	48	14.2 (11.0 to 17.4)	48	19.1 (15.8 to 22.5)	-9.078	≤0.001	-0.43
**Previous/current treatment**							
Previous/current treatment	82	13.2 (11.1 to 15.2)	81	17.7 (15.6 to 19.7)	-5.865	≤0.001	-0.47
No previous/current treatment	114	12.4 (10.7 to 14.1)	112	17.5 (15.6 to 19.3)	-5.458	≤0.001	-0.52
**Personality disorder screening**							
Positive screening, SAPAS>2	59	13.6 (11.4 to 15.8)	67	19.4 (16.8 to 21.9)	-7.167	≤0.001	-0.57
No positive screening, SAPAS≤2	137	11.4 (9.9 to 12.9)	126	16.8 (15.1 to 18.5)	-5.366	≤0.001	-0.60
**Anxiety trial**							
		**Collaborative care**		**Consultation liaison**			
	**N**	**Mean BAI (95% CI)**	**N**	**Mean BAI (95% CI)**	**Difference (Est)**	***P***	**Cohen's d**
**Primary anxiety diagnosis**							
Generalized anxiety disorder	45	13.4 (11.3 to 15.6)	45	17.7 (15.0 to 20.5)	-2.609	0.046	-0.51
Panic disorder/agoraphobia	72	15.1 (12.8 to 17.4)	71	17.7 (15.7 to 19.7)	-3.038	0.026	-0.28
Social anxiety disorder	26	16.5 (13.4 to 19.6)	26	19.3 (15.6 to 23.1)	-2.751	0.152	-0.33
Obsessive-compulsive disorder	8	16.3 (9.2 to 23.3)	9	16.3 (13.6 to 19.0)	-0.533	0.862	-0.004
**Previous/current treatment**							
Previous/current treatment	73	14.9 (13.0 to 16.8)	71	19.6 (17.2 to 22.0)	-3.785	0.004	-0.50
No previous/current treatment	78	14.9 (12.9 to 16.9)	80	16.4 (14.9 to 17.9)	-2.022	0.063	-0.19
**Personality disorder screening**							
Positive screening, SAPAS>2	49	15.2 (13.3 to 17.1)	56	19.3 (16.8 to 21.7)	-3.506	0.007	-0.49
No positive screening, SAPAS≤2	102	14.6 (12.7 to 16.4)	95	16.7 (15.0 to 18.5)	-3.096	0.007	-0.25

Estimates are based on imputed data.

Figure S1. Differences between groups in the depression trial for depression symptoms (BDI-II), anxiety symptoms (BAI), functional level (SDS) and patient satisfaction (CSQ-8)

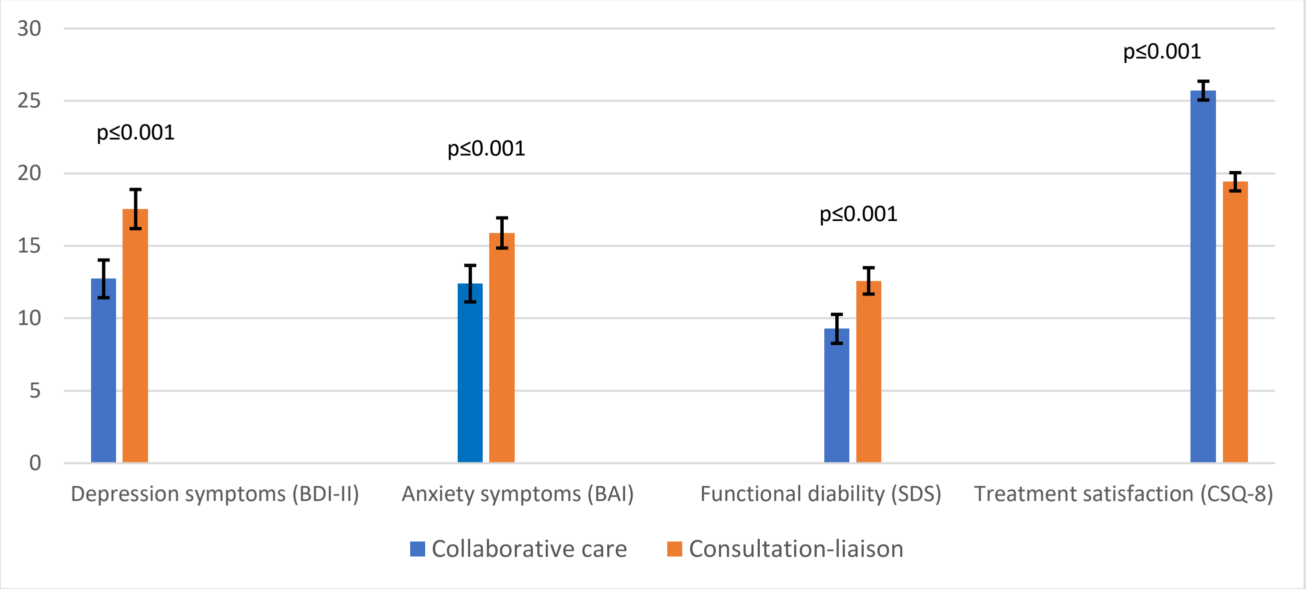

Figure S2. Differences between groups in the anxiety trial for anxiety symptoms (BAI), depression symptoms (BDI-II), functional level (SDS) and patient satisfaction (CSQ-8)

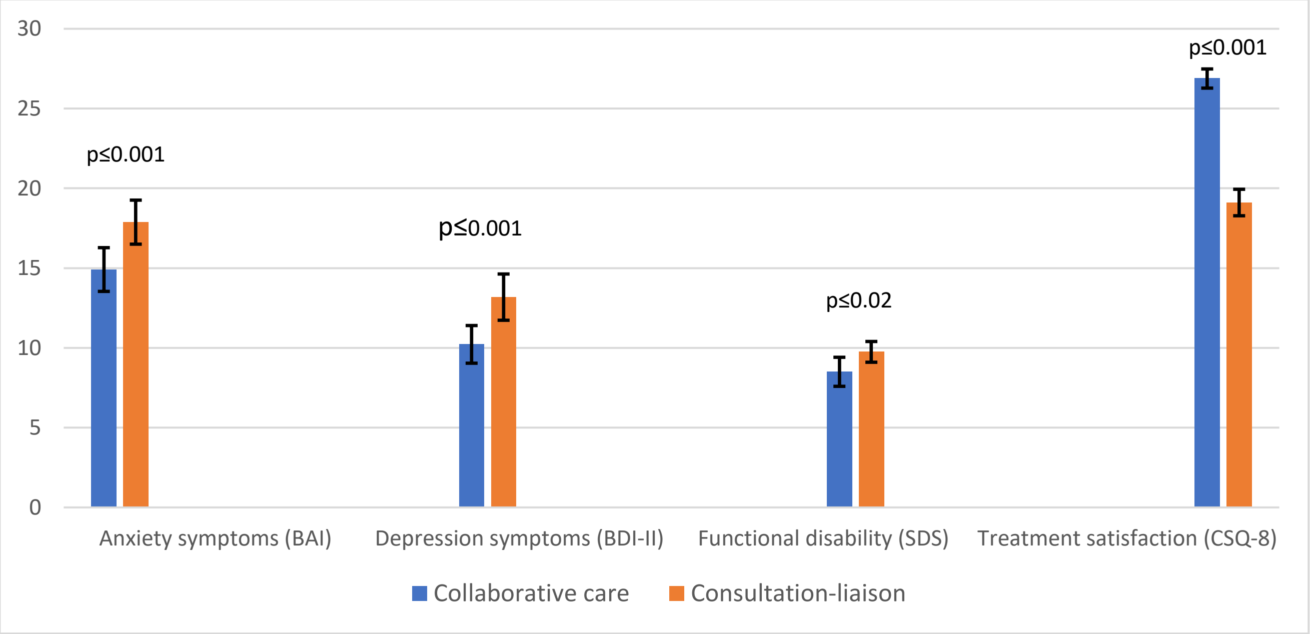

Supplement: Supplementary file 1 [file S0007125023000776sup001.pdf]
